# Discrete strategies to reduce intake of discretionary food choices: a scoping review

**DOI:** 10.1186/s12966-016-0380-z

**Published:** 2016-05-06

**Authors:** Jessica A. Grieger, Thomas P. Wycherley, Brittany J. Johnson, Rebecca K. Golley

**Affiliations:** School of Pharmacy and Medical Sciences, University of South Australia, Adelaide, Australia; School of Health Sciences, University of South Australia, Adelaide, Australia; School of Pharmacy and Medical Sciences, Sansom Institute for Health Research (Public Health Concentration), University of South Australia, Adelaide, Australia

**Keywords:** Discretionary choices, Diet quality, Randomized controlled trials, Review, Energy dense, Nutrient intake

## Abstract

On a population level, dietary improvement strategies have had limited success in preventing the surge in overweight and obesity or reducing risk factors for chronic disease. While numerous multi-component studies have examined whole-of-diet strategies, and single component (i.e. discrete) dietary intervention strategies have targeted an increase in core foods (e.g. fruits, vegetables, dairy), there is a paucity of evidence on the effectiveness of dietary intervention strategies targeting a decrease in discretionary choices. The aim of this review was to identify dietary intervention strategies that are potentially relevant to reducing intake of discretionary choices in 2–65 year olds. A scoping review was carried out to map the literature on key discrete dietary intervention strategies that are potentially applicable to reducing discretionary choices, and to identify the targeted health/nutrition effects (e.g. improve nutrient intake, decrease sugar intake, and reduce body weight) of these strategies. Studies conducted in participants aged 2–65 years and published in English by July 20, 2015, were located through electronic searches including the Cochrane Library, Medline, Embase, CINAHL, and Scopus. Three thousand two hundred and eighty three studies were identified from the search, of which 44 met the selection criteria. The dietary intervention strategies included reformulation (*n* = 13), substitution (*n* = 5), restriction/elimination (*n* = 9), supplementation (*n* = 13), and nutrition education/messages (*n* = 4). The key findings of the review were: restricting portion size was consistently beneficial for reducing energy intake in the acute setting; reformulating foods from higher fat to lower fat could be useful to reduce saturated fat intake; substituting discretionary choices for high fibre snacks, fruit, or low/no-calorie beverages may be an effective strategy for reducing energy intake; supplementing nutrient dense foods such as nuts and wholegrain cereals supports an improved overall diet quality; and, a combination of permissive and restrictive nutrition messages may effectively modify behavior to reduce discretionary choices intake. Longer-term, well-controlled studies are required to assess the effectiveness of the identified dietary strategies as interventions to reduce discretionary choices intake.

## Background

The global prevalence of overweight and obesity, and associated chronic health conditions continues to increase [1]. Dietary recommendations for weight management and chronic disease prevention, including increasing fruit and vegetable intake and decreasing intake of added sugar, saturated fat and salt [2], have failed to be successfully adopted by the majority of the western population [3–5]. In Australia, only 6 % of adults consume an adequate intake of fruit and vegetables [6] and only 69 and 36 % of children (aged 5–11 years) consume at least two and three daily serves of fruit and vegetables, respectively [7]. In the UK only 8.5 % of adolescents and 30 % of adults meet the recommended serves of fruit and vegetables [5]. Similarly in the US, only 19 % of the population meet the minimum serves of fruit and vegetables [4]. Discretionary choices are foods or beverages high in saturated fat, added sugars, or salt, such as crisps, sugar sweetened beverages, sweet biscuits, cakes and desserts, sweet and savory pastries and processed meats [8]. In Australia, discretionary choices currently contribute around 36 % of energy intake in 2–13 year olds and 30–40 % of energy intake in those aged ≥14 years [3]. According to US data, 86 % of the population consume more than the recommended limit of discretionary choices [4]. National dietary intake data from the UK also shows mean intake of saturated fat, sodium and added sugars are in excess in all age groups (with the exception of sodium in girls aged 7–10 years) [5]. Research in children has indicated that discretionary choices may displace core foods such as fruit, vegetables, dairy, lean meats, and whole grains [9, 10]. Reducing the current intake of discretionary choices will reduce the risk of nutrient deficiencies, obesity and associated chronic disease [8].

While the impetus to reduce discretionary choice intake is clear, the interventions needed to achieve this change are uncertain. The effectiveness of multi-component dietary interventions (e.g. changing whole-of-diet) or discrete dietary intervention strategies targeting an *increase* in core foods (e.g. fruit and vegetable or dairy intake) have been widely researched; however they have had little success in preventing the surge in overweight and obesity [11–13] as well as reducing risk factors for chronic disease [14, 15]. In contrast, there is a paucity of evidence on the effectiveness of dietary intervention strategies targeting a *decrease* in discretionary choices. Increasing our understanding of dietary strategies that are potentially relevant to *decreasing* discretionary choices intake will inform the design of next generation interventions which are needed to prevent overweight and obesity and/or reduce chronic disease risk factors. Therefore, the aim of this scoping review is to identify dietary intervention strategies that are potentially relevant to *reducing* intake of discretionary choices in 2–65 year olds. We will explore evidence from interventions targeting discretionary choices and examining dietary intervention strategies that have been applied in the context of core foods but could be applied in the context of reducing discretionary choice intake.

## Methods and approach

A scoping review was conducted based on key phases detailed by Arksey et al [16] with the aim of mapping the literature on key discrete dietary intervention strategies that are potentially applicable to reducing discretionary choices, and their targeted health/nutrition effects (e.g. improve nutrient intake, decrease sugar intake and reduce body weight) in 2–65 year olds. According to Daudt et al., a scoping review aims to “map the literature on a particular topic or research area and provide an opportunity to identify key concepts; gaps in the research; and types and sources of evidence to inform practice, policymaking, and research” [17]. The framework for conducting this scoping review was based on key phases detailed by Arksey et al [16]: i) identification of the research question to be addressed; ii) identification of studies relevant to the research question; iii) selection of studies to include in the review; iv) charting of information and data within the included studies; and v) collating, summarizing and reporting results of the review.

### Search strategy

The search strategy and procedure were guided by the PRISMA statement [18]. Potential studies were located through electronic searches (Cochrane Library, Ovid [Medline and Embase], EbscoHost [CINAHL], and Scopus). Limits were set to age 2–65 years, English language, and publications released up to and including July 20, 2015 (i.e. the search date). Search terms and MeSH headings in the title, abstract, and index terms were initially identified in Medline and subsequent key words were used for the remaining databases (Appendix 1). An academic librarian assisted with the development of MeSH headings, key words, and conduct of the electronic database search.

**Fig. 1:**
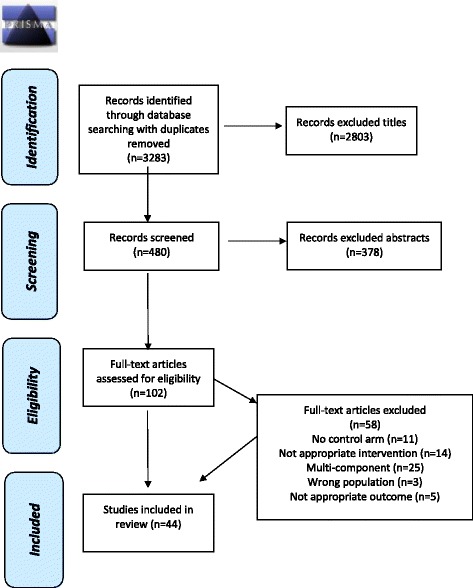
PRISMA Flow chart of included studies. Flow chart indicates 3283 articles were retrieved (with removal of duplicates), followed by exclusion of 2803 articles based on irrelevant titles and exclusion of 378 abstracts. One hundred and two full text articles were examined in which 58 full text articles were excluded; leaving 44 studies included in the review

Identified studies were assessed for inclusion by the primary author (JAG). Following removal of duplicates, articles were examined for eligibility based initially on titles and abstracts, followed by a full-text appraisal of all remaining articles (Fig. 1). Reasons for full text exclusion were documented.

### Study selection

Included participants were children and adults aged 2–65 years who were generally healthy and without chronic disease. Included studies were randomized controlled trials (RCT) or comparator group studies that evaluated strategies to reduce discretionary food intake (or core foods, e.g. dairy products, with an approach that could be applied to discretionary choices) with the aim to improve nutritional intake or health status. Strategies included those pertaining to dietary manipulation with a focus on reformulation (e.g. low fat vs. high fat products), substitution (e.g. replacing biscuits with fruit), restriction/elimination (e.g. reducing portion size), and supplementation (e.g. including specific foods/beverages in the diet). Chronic and acute studies were included: chronic dietary intervention studies were those where participants would consume the food/beverage daily for a number of weeks and dietary intake measured post intervention; acute studies were those where the food/beverage was consumed only once, or once per week over a few weeks, and the impact of subsequent food/calorie intake was measured. Strategies utilizing nutrition education/messages were also included where participants were provided information/strategies/messages on food for any defined period, to alter food intake. Exclusion criteria were studies in pregnant or lactating women or clinical populations that require strictly modified diets (e.g. those with celiac disease); studies related to binge/disordered/restrained eating; studies assessing results of only the intervention or comparator group either following the RCT or as follow-up post-RCT completion; studies assessing food labelling and media advertising (e.g. through computer games or television adverts); and studies that did not report on nutritional or food intake or body weight.

### Data collation and reporting of results

Data was extracted on the study aims, intervention, outcome measurement and main results (see results Tables 1, 2, 3, 4 and 5) and summaries were developed. Unlike a systematic review which synthesizes and weights studies according to level of evidence, a scoping review presents findings in a narrative way. We based our narrative synthesis on recommendations by Arksey et al. [16] and Popay et al. [19]. That is, we firstly gave attention to the numbers of studies reporting on each discrete intervention type (e.g. substitution, reformulation, and elimination), a description of the intervention, and whether it was effective at producing a significant change in the study’s main outcome. This allowed us to understand what the most common themes emerging from the identified strategies were, map the strategies back to our aim, and to have a better understanding on their effectiveness and research gaps. Together, these data formed the basis of the scoping review summary.

**Table 1:** Description of included reformulation studies

Reference	Study aims	Intervention type, comparator and duration	Outcome measurement	Main results
*Chronic studies*				
Gatenby SJ, 1995 USA	To assess the nutritional implications of the purchase and consumption of reduced-fat foods at home in normal-weight, free-living consumers. Ages (mean age 40 yrs) Total n completed = 29	Randomized trial for 6 weeks. 1) Experimental: Use reduced-fat foods *ad-libitum* in place of traditionally high-fat foods. 2) Control: Habitual diet.	1) 4 d weighed food diaries 2) Body weight	1) The low fat group reduced their % of energy from fat vs. control at 6 weeks (mean ± SEM: 38.3 ± 1.8 % to 30.4 ± 1.7 % vs. 37.9 ± 1.9 % to 39 ± 4 %, *P* = 0.001). 2) The low fat group increased the % of energy from protein (17 ± 11 % E vs. 15 ± 0.5 % E, *P* = 0.06) and carbohydrate (49 ± 2 % E vs. 46 ± 2 % E, *P* = 0.019) vs. control. 3) NS in mean ± SEM total energy intake between experimental and control groups (1939 ± 598 kJ vs. 1887 ± 417 kJ, *P* = 0.63). 4) NS in mean ± SEM sugar intake between experimental and control groups (109 ± 11 g vs. 99 ± 15 g, *P* > 0.05). 5) The low fat group lost 1.1 kg (*P* < 0.001) while the control group had a non-significant gain of 0.4 kg (mean ± SEM baseline to final weight in experimental: 74.6 ± 4.5 kg to 73.5 ± 4.3 kg; control: 65.4 ± 2.9 kg to 65.7 ± 2.8 kg).
Gatenby SJ, 1997 USA	To expand and extend the previous study (above) while also contrasting the effects of fat and sugar replacement. Ages: 18-50 yrs Total n completed = 65 females	Randomized trial for 10 weeks. 1) Reduced fat: Use reduced-fat foods *ad-libitum* in place of habitually consumed foods with traditional composition. 2) Reduced sugar: Use reduced-sugar foods *ad-libitum* in place of habitually consumed foods with traditional composition. 3) Control: Maintain usual diet.	4 d weighed food diaries	1) NS overall main or interactive effects of group for energy intake, sugar intake; however all groups reduced their sugar intake (data not reported in paper). 2) Compared with the reduced sugar and control groups, the reduced fat group reduced their fat intake significantly during the intervention period from ~37 % E from fat at baseline to 33 % E from fat at week 10 (*P* *=* 0.017).
Gunther CW, 2005 USA	To determine the effects of a 1-yr intervention of dairy calcium on changes in body weight and fat mass in healthy women, aged 18–30 yrs. Ages: 18-30 yrs (mean 20 yrs) Total n completed = 41 in control, 44 in medium, 48 in high dairy	Randomized controlled trial for 1 year. 1) Control: Continue established dietary intake. 2) Medium dairy: Substitute dairy products to achieve calcium intake of approximately 1000-1100 mg/d and maintain isocaloric intake. 3) High dairy: Substitute dairy products to achieve calcium intake of 1300-1400 mg/d and maintain isocaloric intake. Groups 2) and 3) instructed to increase intake of daily calcium by substituting dairy products rich in calcium, with an emphasis on non-fat and low-fat milk.	3 d food records to assess calcium intake	No significant change in 1 y body weight between groups (control: 0.8 ± 2.8 kg, medium-dairy group: 0.7 ± 3.0 kg, high-dairy group: 1.5 ± 4.1 kg).
Golley RK, 2012 Australia	To undertake a secondary analysis to evaluate the impact of changing children’s dairy food choices, in terms of fat type, on children’s total food intake and examine the contribution of dairy foods to energy and fat intake relative to other food groups Ages: Families comprised one parent and their healthy children aged 4-13 yrs Total n completed = 137 children	Cluster randomized controlled trial (secondary analysis) for 24 weeks. 1) Parents asked to change dairy foods they purchased for the family and offered to their children (i.e., replacing regular- with reduced- or low-fat varieties). 2) Individualized advice: Encouraged to replace screen-based activities with other sedentary activities, to try to avoid an increase in physical activity.	24 h recall	1) Week 12: Children in the intervention group consumed 1.0 (0.6, 1.3) servings per day more reduced-fat dairy vs. control group (*P* < 0.0001) 2) Week 24: Reduced fat dairy intake was higher in the intervention vs. control group by 0.8 (0.5, 1.1) servings per day, *P* < 0.0001. 3) Week 24: Contribution of total dairy to total saturated fat intake was significantly lower in the intervention group vs. the comparison group: 10 ± 11 % vs. 20 ± 14 %, *P* < 0.01.
Ebbeling C, 2006 USA	To test the hypothesis that a simple environmental intervention will significantly decrease SSB consumption and BMI among adolescents. Ages: 13-18 yrs Total n completed = 103	Randomized, controlled pilot study for 25 weeks. 1) Received weekly home deliveries of noncaloric beverages for 25 weeks (4 × 360 ml or 12 fl oz per referent serving). The target number of delivered servings was ~5 units/week. 2) Control: Continue usual beverage consumption habits.	1) 2x 24 h dietary recalls 2) Physical activity recall	1) NS in mean ± SEM BMI between intervention and control (0.07 ± 0.14 kg/m^2^ vs. 0.21 ± 0.15 kg/m^2^(Δ -0.14 ± 0.21 kg/m^2^, *P* > 0.05). 2) Mean change in energy intake was lower in the intervention vs. control group (-1201 ± 836 kJ vs. -185 ± 94 kJ, *P* < 0.001). 3) Intervention group increased non-caloric beverage intake vs. control (396 ± 493 ml/d vs. 78 ± 523 ml/d, *P* = 0.002).
Raben A, 2002 Denmark	To investigate the effect of long-term supplementation with drinks and foods containing either sucrose or artificial sweeteners on *ad-libitum* food intake and body weight in overweight subjects. Ages: 20-50 yrs (mean 33 yrs in sucrose vs. 37 yrs in sweetener group) Total n completed = 41	Parallel design with 2 intervention groups for 10 weeks in overweight adults. 1) Received supplemental drinks and foods containing sucrose (~70 % of the sucrose came from drinks and ~30 % came from solid foods to reach a sucrose intake of ~2 g/kg body weight; foods/drinks included soft drinks, fruit juices, yoghurt, ice-cream). 2) Received similar drinks and foods containing artificial sweeteners (in similar amounts to sucrose group).	1) 7 d dietary records for energy and nutrient intakes 2) 7 d diaries for monitoring hunger, fullness, palatability of the food, and wellbeing)	1) Energy intake from the sucrose supplements was ~3 times higher than that from the sweetener supplements (3349 ± 66 kJ vs. 963 ± 44 kJ, diet effect *P* < 0.0001). 2) Higher amounts of total carbohydrate (%E) consumed from the sucrose vs. sweetener supplements (89 ± 0 % vs. 52 ± 2 %, *P* < 0.05). 3) Total daily energy intake higher in the sucrose vs. sweetener group (11452 ± 551 kJ vs. 8656 ± 416 kJ, *P* = 0.03 diet × time interaction). 4) Total daily fat intake (% E) was lower in the sucrose vs. sweetener group (29 ± 1 % vs. 33 ± 1 %, P = 0.005 diet × time interaction). 5) Body weight increased in the sucrose group and decreased in the sweetener group (+1.6 kg vs. -1.0 kg, *P* < 0.0001).
*Acute studies*				
Wilson J, 2000 USA	1) To examine the eating behavior of a large number of preschool children offered chocolate-flavored or plain milk at lunch. 2) To examine whether aspartame-sweetened (sugar-free) chocolate milk also induced an increase in energy intake during the meal Ages: 1.5–5.5 yrs Total n completed = 135	Randomized controlled trial Four different menus served six times during a 12-week period, each menu being presented twice with each of three test beverages: 1) Plain milk (18.1 kcal/oz). 2) Sucrose-sweetened chocolate milk (29.4 kcal/oz). 3) Aspartame-sweetened chocolate milk (18.6 kcal/oz).	Weighed portions	1) The type of milk beverage served had no significant effect on the consumption of other food items offered at that meal. 2) Children consumed more energy (134-155 kcal vs. 48-66 kcal, *P* < 0.05) during meals in which sucrose-sweetened chocolate milk was served.
Harris J, 2011 USA	To test (1) whether children will consume low-sugar RTEC and (2) the effects of serving high- versus low-sugar cereals on the consumption of cereal, refined sugar, fresh fruit, and milk. Ages: 5-12 yrs (mean 8.4 yrs) Total n completed = 91	Randomized trial. 1) High-sugar RTEC (3 cereals offered, 11-12 g of sugar per serving, 28-33 g) 2) Low-sugar RTEC (3 cereals offered, 1-4 g of sugar per serving, 28-33 g) Children poured their own cereal. Each place-setting contained an 8-oz carton of 1 % low-fat milk (245 g), a small container of orange juice (182–197 g), and bowls of precut strawberries (140 g) and bananas (111 g). A bowl of sugar packets and additional orange juice and milk cartons were placed in the middle of each table.	1) Sugar and calorie content obtained from nutrition facts panels on the cereals 2) US Department of Agriculture National Nutrient Database for Standard References for other breakfast items.	Children in the high sugar condition: 1) Consumed more RTEC vs. children in low sugar condition (mean (SD): 61.3 (39.1) vs. 34.6 (24.3), *P* < 0.001). 2) Consumed more refined sugar from RTEC vs. low sugar RTEC (22.9 (14.4) vs. 2.9 (2.6), *P* < 0.001). 3) Consumed more refined sugar overall (from RTEC and added sugar: 24.4 (15.1) vs. 12.5 (11.7), *P* < 0.001). Children in the low sugar condition: 1) Added more sugar to their RTEC vs. children in the high sugar condition (9.6 (10.7) vs. 1.4 (2.8), *P* < 0.001) 2) NS between groups in other foods consumed (e.g. milk, fruit, orange juice).
Vitaglione P, 2010 Italy	To investigate new type of biscuit containing 5.2 % barley beta-glucan and its effect on appetite moods and food intake. Ages: mean 18 yrs Total n completed = 20	Five sessions in which subjects participated in a randomized order and with a week frequency. Midmorning snack: 1) 628 kJ preload of barley beta-glucan-enriched biscuit. 2) 1884 kJ preload of barley beta-glucan-enriched biscuit (g/100 g: Energy: 1653 kJ; Protein: 6.1 g; Fat: 13.9 g; Carbohydrate: 61.4 g; Total dietary fibre: 12.6 g; soluble fibre: 8.3 g; beta-glucan: 5.2 g; insoluble fibre: 4.3 g). 3) Control biscuit (g/100 g: Energy: 1716 kJ; Protein: 8.6 g; Fat: 13.6 g; Carbohydrate: 63.2 g; Total dietary fibre: 2.5 g; soluble fibre: 1.2 g; beta-glucan: 0 g; insoluble fibre: 1.3 g).	Not reported	No effect of food intake between any of the snack groups.
Johnstone A, 2000 UK	To examine the effects of 1) ingesting mandatory snacks vs. no snacks; 2) the composition of isoenergetically-dense snacks high in protein, fat or carbohydrate, on food intake and energy intake in eight men with *ad-libitum* access to a diet of fixed composition. Ages: mean age 27 yrs Total n completed = 8 men	Subjects were required to consume three mandatory isoenergetically dense snacks of the same energy content at three fixed-time points; served as a salad, pate and a yoghurt-style drink. 1) High protein (total 1.88 MJ protein from snacks). 2) High carbohydrate (total 1.93 MJ carbohydrate from snacks). 3) High fat (total 1.92 MJ fat from snacks). 4) No snacks.	Not reported	1) Total daily energy intake (inclusive of snacks) was not significantly different across treatments (*F*(3,21) 0.55; *P* *=* 0.654). 2) NS in mean ± SE weight change between groups (high protein: Δ +0.48 ± 0.06 kg; high carbohydrate: Δ +0.33 ± 0.05 kg; no snacks: Δ -0.16 ± 0.06 kg; high fat: Δ -0.03 ± 0.04 kg, *P* > 0.05).
Ortinau LC, 2013 USA	To evaluate the impact of a higher-protein afternoon snack on appetite control, delays in eating initiation, and subsequent energy intake compared to an isocaloric normal protein snack in healthy women. Ages: mean 27 yrs Total n completed = 32	Randomized crossover-design. Afternoon yogurt snacks containing: 1) Normal protein yoghurt (5 g protein/170 g serve). 2) Higher-protein Greek yoghurt (14 g protein/170 g serve).	Visual analogue scales	1) NS *ad-libitum* dinner intake between the normal protein and high protein snacks (686 ± 33 kcal vs. 709 ± 34 kcal, *P* = 0.324).
Leahy KE, 2008 USA	To investigate the effects of reducing the energy density of a popular and familiar entrée—macaroni and cheese—on children’s energy intake at lunch. Ages: 2-5 yrs (mean 3.9 yrs) in a university day-care facility Total n completed = 77	Within-subjects crossover design: All children received a standard breakfast and then a manipulated (energy density) entrée (macaroni and cheese, 300 g) 1 day per week for 7 weeks. Included in the meal was broccoli, applesauce, and milk. 1) Higher-energy-density entrée had 2.0 kcal/g. 2) Lower-energy-density entrée had 1.4 kcal/g.	Weighed food before and after eating	1) Decreasing the energy density of the macaroni and cheese by 30 % resulted in a 25 % (72.3 ± 8.3 kcal) decrease in energy consumed from the macaroni and cheese (*P* < 0.05) and total lunch intake by 18 % (*P* < 0.0001). 2) Compared with the higher-energy-density macaroni and cheese, children consumed an additional 10.1 ± 4.2 g of the lower-energy-density macaroni and cheese (*P* < 0.05).
Osterholt KM, 2007 USA	To test how short-term ad libitum intake is affected by variations in the air content of a snack food Ages: 19-45 yrs (mean 27 yrs) Total n completed = 28	Cross-over design with repeated measures within subjects. Subjects consumed a snack on 4 separate afternoons at least 3 days apart (differing in amount of incorporated air). Both snacks had an energy density of 5.7 kcal/g and contained 56 % of energy as fat. Snacks differed slightly in sodium content (less-aerated: 1.0 % of weight; more-aerated: 1.3 % of weight). Subjects were served the same volume of each snack (approximately 1250 ml), but received 55 % less weight and energy when served the more-aerated snack rather than the less-aerated snack.	Weighed food before and after eating	1) Subjects consumed a mean of 70 ± 17 fewer kcal of the more-aerated snack than the less-aerated snack, equivalent to a 21 % decrease in energy intake (*P* = 0.0003). 2) By volume, consumption of the more-aerated snack was 239 ± 24 ml greater, equivalent to a 73 % increase in the volume consumed (*P* < 0.0001). 3) NS in subsequent snack intake (data not reported in paper).

*BMI *body mass index, *n* number of participants, *NS* not significant, *RTEC* ready to eat cereal, *SD* standard deviation, *SE* standard error, *SEM* standard error of the mean, *SSB* sugar sweetened beverage, *yrs* years of age, *%E* percentage of energy

**Table 2:** Description of included substitution studies

Reference	Study aims	Intervention type, comparator and duration	Outcome measurement	Main results
*Chronic studies*				
Brauchla M, 2013 USA	To determine the effect of introducing two high-fiber snacks per day on gastrointestinal function as well as nutrient and food group intake in healthy children ages 7–11 yrs old Ages: 7-11 yrs Total n completed = 80	Cluster randomized-controlled prospective community-based intervention for 8 weeks. 1) Consume two high-fiber snacks per day (7 d/week, 10-12 g/d fiber). Each child also offered 8-ounce carton of skim milk at each snack occasion to provide fluids to prevent gastrointestinal distress. 2) Control: Usual snacks.	2 × 24 h dietary recalls	1) NS in mean (SD) macronutrient, fibre or micronutrient intakes between groups at the end of the intervention. 2) NS in mean (SD) wholegrain intake between groups at the end of the intervention (0.77 (1.0) servings/d vs. 0.56 (0.76) servings/d, *P* > 0.05). 3) Intake of mean (SD) total grains (6.30 (2.28) servings/d vs. 5.45 (1.58) servings/d), and sweets (0.55 (0.64) servings/d vs. 0.33 (0.37) servings/d, all *P* < 0.05) was higher in the intervention vs. control group.
Zaveri S, 2009 UK	To investigate the effect of incorporating a novel type of snack (almonds) and a conventional snack (cereal bars), on eating frequency, hunger rating, total energy intake, fasting glucose, insulin and lipid levels and anthropometric measures over a 12 week period in a sample of overweight Scottish men Ages: 25-50 yrs Total n completed = 36	Randomized trial for 12 weeks 1) Two cereal bars (30 g; high in carbohydrate, total 44 g carbohydrate). 2) Two packets of almonds (28 g; high in protein, total 11.8 g). 3) Control: No snacks and asked to continue habitual eating pattern.	1) 4 d unweighed diet diaries 2) Anthropometry	1) NS in intake of energy, protein, fat or sugar intake between groups after the intervention (*P* > 0.05). 2) NS between groups in body weight or waist:hip ratio (*P* > 0.05).
*Acute studies*				
Flood J, 2006 USA	To examine the impact of increasing beverage portion size on beverage and food intake. Ages: 18-45 yrs Total n completed = 33	Cross-over study. Subjects came to the laboratory to eat lunch once a week for 6 weeks, for a total of six test sessions. Subjects ate a standard breakfast, and then lunch differed in portion size of beverage (same food). One of three beverages served in one of two portion sizes (360 g or 540 g): 1) Regular cola (150 cal/260 g; 250 kcal/540 g). 2) Diet cola (0 cal). 3) Water (0 cal).	Weighed food before and after eating	1) Subjects consumed more energy from the caloric beverage (regular cola) when served the large portion (151 ± 8 kcal) vs. the small portion (128 ± 4 kcal, *P* < 0.05). 2) Subjects consumed more water (380 ± 10 g) than regular cola (335 ± 11 g) and diet cola (298 ± 12 g), and more regular cola than diet cola (all *P* < 0.0001). 3) Food intake at lunch did not differ by either type or portion size of the beverage served (*P* > 0.05).
Rolls BJ, 2010 USA	To investigate the effects on food and energy intakes of varying the portion size and energy density of a vegetable that was added to a meal or substituted for other foods. Ages: 20-45 yrs (mean 27 yrs) Total n completed = 48 in the substitution study	Crossover design with repeated measures. Two studies: In both studies, a midday meal of a vegetable, grain, and meat served to participants once a week. Across the meals, the vegetable was increased in portion size (180, 270, or 360 g) and reduced in energy density (0.8 to 0.4 kcal/g). Substitution study: as the vegetable portion was increased, the amounts of the meat and grain decreased equally (i.e. the total amount of food served at the meal did not change).	Weighed food before and after eating	1) Increasing the portion of the vegetable from 180 to 270 g increased vegetable intake in both studies by a mean ± SE of 34 ± 4 g, *P* < 0.0001, equivalent to ~ ½ serving. 2) Doubling the portion of the vegetable (180-360 g) increased vegetable intake by 60 ± 5 g (49 ± 4 %), *P* < 0.0001, equivalent to ~ ¾ serving. 3) Reducing the energy density of the vegetable led to a small decrease in vegetable consumption (9 ± 3 g, P = 0.002). 4) Substitution study: intakes of the meat and grain decreased as the portion of the vegetable increased from 180 to 270 g; significant decrease in energy intake from energy-dense meat and grain as portion sizes decreased (40 ± 10 kcal; *P* < 0.0001).
Patel BP, 2013 Canada	To examine appetite and energy intake following *ad-libitum* consumption of an afterschool snack of raisins, grapes, potato chips, and chocolate chip cookies in children 8 to 11 yrs. Ages: 8-11 yrs (mean 10 yrs) Total n completed = 26	Within-subjects repeated measures design. Children were given: 1) Grapes (301 g) 2) Raisins (65 g) 3) Potato chips (38 g) 4) Chocolate chip cookies (45 g) to consume (within 15 min) as an afternoon snack.	Weighed food before and after eating	1) Mean ± SEM snack intake was lowest after raisins (228 ± 21 kcal) and grapes (177 ± 17 kcal) compared to potato chips (413 ± 20 kcal), however cookies was highest (505 ± 32 kcal, *P* <0.001). 2) Cumulative food intake was lowest after raisins (1099 ± 21 kcal) and grapes (1049 ± 17 kcal; *P* < 0.001) compared to potato chips (1284 ± 20 kcal), however, cookies was highest (1376 ± 32 kcal; *P* <0.001). 3) Neither energy density nor volume predicted the effect of the snack on cumulative energy intake (Energy densities of raisins (3.04 kcal/g), chips (5.58 kcal/g), and cookies (4.68 kcal/g) were higher than for grapes (0.69 kcal/g), but grapes and raisins had similar effects on cumulative food intake.

*n* number of participants, *NS* not significant, *SD* standard deviation, *SEM* standard error of the mean, *yrs* years of age

**Table 3:** Description of included restriction/elimination studies

Reference	Study aims	Intervention type, comparator and duration	Outcome measurement	Main results
*Acute studies*				
Rolls BJ, 2004 USA	To determine whether the meal energy intake was minimized by consuming one of the salads as a first course, or by omitting the first course completely. Ages: 19-45 yrs (mean 26 yrs) Total n completed = 42 women	Within-subjects crossover design with seven experimental conditions. Once per week for 7 weeks, subjects came to the laboratory to eat lunch (salad and pasta). At six of the lunches, the first course was one of six different versions of a salad that varied in energy density and portion size; subjects were required to consume the salad in full. 1) Energy density 0.33 kcal/g (a- 150 g [equivalent to 1.5 cups] and b- 300 g [equivalent to 3 cups]) 2) Energy density 0.67 kcal/g (a- 150 g and b- 300 g) 3) Energy density 1.33 kcal/g (a- 150 g and b- 300 g) In the control condition, no food was served for the first course.	Weighed food before and after eating	1) Doubling the portion size of the salad (300 g vs. 150 g) reduced intake of the pasta (98 ± 30 kcal, *P* < 0.0001). 2) Eating the low-energy-dense salad decreased meal intake by 7 % (64 ± 26 kcal) for the small portion and 12 % (107 ± 29 kcal) for the large portion. 3) Consuming the high-energy-dense salad increased meal intake by 8 % (71 ± 27 kcal) for the small portion and 17 % (145 ± 22 kcal) for the large portion). 4) In comparison with having no first course, eating the low-energy-dense salad decreased meal intake by 7 % (64 ± 26 kcal) for the small portion and 12 % (107 ± 29 kcal) for the large portion (*P* < 0*.*0001).
Savage BJ, 2012 USA	To examine the effect of varying entrée portions on children’s *ad-libitum* energy intake of macaroni and cheese and fixed portions of unsweetened applesauce, green beans, and whole-wheat roll served with the entrée Ages: 3-6 yrs Total n completed = 17	Within-subject design with repeated measures. Macaroni and cheese meal and fixed portions of unsweetened applesauce, green beans, and whole-wheat roll served with the entrée (different entrée portions: 100 g, 160 g, 220 g, 280 g, 340 g, 400 g)	Weighed food before and after eating	1) The percentage of the macaroni and cheese eaten decreased significantly as portion size increased (i.e. 95 % of 100 g portion eaten down to 64 % of 400 g portion eaten, *P* < 0.001). 2) Increasing portion size (i.e. 100-400 g) increased children’s intake of the macaroni and cheese (~100 g up to ~ 250 g, *P* < 0.01) 3) Increasing portion size decreased the intake of other foods served with the macaroni and cheese such as fruit and vegetables (~180 g other foods consumed with 100 g macaroni and cheese portion, down to ~100 g of other foods consumed with 400 g macaroni and cheese portion, *P* < 0.0001). 4) There was a 61 % higher energy intake at lunch as portion size increased (*P* < 0.0001).
Fisher J, 2007 USA	To test the effects of portion size and energy density on children’s food and energy intakes at a meal. Ages: 5-6 yrs Total n completed = 53	2 × 2 within-subject factorial design. Each child was seen in 4 conditions differing only in the portion size (329 kcal/250 g vs. 658 kcal/500 g in the 1.3 kcal/g entrée and 460/250 g vs. 920 kcal/500 g in the 1.8 kcal/g) and energy density (1.3 kcal/g vs. 1.8 kcal/g) of a macaroni and cheese entrée served at a dinner meal.	Weighed food intake	1) Children consumed 33 % more of the entrée in the large portion conditions vs. reference conditions (210 ± 11 g vs. 158 ± 11 g, *P* < 0.0001). 2) Children consumed 33 % more energy (332 ± 19 kcal vs. 249 ± 19 kcal; *P* < 0.0001) from the entrées when served either the larger or the more energy-dense entrées vs. reference versions. 3) Total energy intake consumed at the meal was ~15 % higher when the large vs. reference portion entrées were served (548 ± 19 kcal vs. 478 ± 19 kcal, *P* < 0.001).
Ebeling CB, 2007 USA	To determine whether reducing portion sizes and slowing eating rate, to attenuate gorging, would decrease energy intake, during a fast food meal. Ages: 13-17 yrs (mean 15 yrs) Total n completed = 20	Feeding study with cross-over design. 1) Meal (chicken nuggets, French fries, and cola) presented as 1 large serving at a single time point: (Control A). 2) Same meal as above portioned into 4 smaller servings presented at a single time point (Condition B). 3) Same meal as above portioned into 4 smaller servings presented at 15-minute intervals (Condition C).	Weighed food before and after eating	1) NS in mean ± SEM energy intake between conditions (A: 7350 ± 496 kJ; B: 7287 ± 491 kJ; C: 7333 ± 487 kJ).
Looney SM, 2011 USA	To investigate the impact of portion size and energy density on intake, both grams and kilocalories, of snacks in preschool-aged children. Ages: 2-5 yrs (mean 3.9 yrs) Total n completed = 35	A 2 × 2 crossover design (within-subject factors of portion size and energy density). Small portion size was 150 g (lower-energy dense = 64.5 kcal of apple sauce; higher-energy-dense = 178.5 kcal of chocolate pudding). Large portion size was 300 g (lower-energy-dense = 129 kcal of apple sauce; higher-energy-dense = 357 kcal of chocolate pudding). The same lunch menu used for all intervention days.	1) Weighed food before and after eating 2) Anthropometry	1) More calories were consumed with increasing portion size (small vs. large portion size: 84.2 ± 30.8 kcal vs. 99.0 ± 52.5 kcal, *P* < 0.05).
Marchiori D, 2011 Belgium	To examine the effect of modifying food-item size of snack foods on subsequent portion and energy intake in an individualized and free-consumption setting. Ages: 18-27 yrs (mean 20 yrs) Total n completed = 33	Randomized between-subjects design with two experimental conditions. 1) Candies left unchanged (10 normal-sized red candies and 10 normal-sized cherry candies). 2) All candies were cut in half (20 half-sized red ribbon candies (2 g each) and 20 half-sized cherry shaped candies (2.5 g each).		1) Participants with the smaller candies consumed the same number of candies vs. those with larger candies (6.2 candies vs. 6.9 candies, *P* > 0.7). 2) Participants offered the larger candies consumed twice as much in gram weight of candies (30.7 ± 18.2 g vs 16.3 ± 20.3 g, P = 0.04), equivalent to a an increase in nearly 60 kcal (109.04 ± 64.5 kcal vs 49.22 ± 57.2 kcal, respectively).
Marchiori D, 2012 Belgium	To examine the effect of modifying small vs. large cookies on children’s food and caloric intake in a typical and familiar eating environment. Ages: Mean age 9 yrs Total n completed = 77	Between-subjects randomized design with 2 experimental conditions: 1) Half the children received 36 half-sized cookies (3.5 g each) 2) Half the children received 18 normal-sized cookies (7 g each).		1) Children offered the smaller cookies consumed more cookies than children offered larger cookies (14.6 cookies vs. 9.2 cookies, *P* < 0.001). 2) Mean energy intake from the large cookies was higher than in children consuming the smaller cookies (342 kcal vs. 274 kcal, *P* < 0.05). 3) Children in the large item condition consumed 25 % more gram weight of cookies vs. children in the small item condition, resulting in an increase of 68 kcal (i.e. 64 kcal vs. 51 kcal, *P* < 0.05).
Stroebele N, 2009 USA	To determine whether or not the portion-controlled packages of snack foods result in less consumption as compared to larger packages when the amount of food provided was held constant. Ages: Mean age 38 yrs Total n completed = 59	Randomized two-period cross-over design for two 7-day study period, with a 1-week washout period. 1) Standard size packages of 10 different snack choices. 2) 100 kcal packages of 10 different snack choices. Participants asked to take the box home and to consume as much and whenever they would like over a 7-day period. The 100 kcal snack package units ranged from 19-26 g per package whereas the standard size package units ranged from 187-369 g.	Weighed food record	Participants consumed ~187 fewer grams of snacks per week when receiving 100 kcal snack packs vs. standard size packages of snacks (*P* < 0.0001).

*n* number of participants, *NS* not significant, *SEM* standard error of the mean, *yrs* years of age

**Table 4:** Description of included supplementation studies

Reference	Study aims	Intervention type, comparator and duration	Outcome measurement	Main results
*Chronic studies*				
Tan SY, 2013 Australia	To determine (1) the acute post-ingestive effects of almond consumption with meals or alone as snacks and (2) the short-term effects of almond consumption on body weight, body composition and indicators of metabolism. Patients at increased risk for type 2 diabetes Ages: 18-60 yrs (mean ~30 yrs) Total n completed = 137	4 week randomized, parallel-arm study. Consumption of 43 g/d almonds with: 1) Breakfast 2) Lunch 3) Morning snack (and nothing else) 4) Afternoon snack (and nothing else) 5) Control: no almonds	1) Anthropometry 2) 24 h dietary recall	1) Despite the additional 250 kcal/day from almonds, daily energy intake in all almond groups was not significantly higher than baseline or the control group. 2) NS in body weight between groups. 3) Almond intake had no effect on the intake of other nutrients at baseline, week 2 or week 4, except for dietary monounsaturated fat and α-tocopherol intake.
Sabate J, 2005 USA	To determine changes in body weight and composition when free-living subjects who are not given additional dietary advice incorporate moderate amounts of walnuts into their diet for 6 months Ages: 30-72 yrs (mean 54.3 yrs) Total n completed = 90	Randomized cross-over field trial with 2x6 month periods. 1) Intervention: Provided walnuts at ~12 % of their daily energy intake (range 28-56 g/d). 2) Control: No diet but asked to refrain from consumption of walnuts for 6 months.	1) Anthropometry 2) 24 h dietary recalls	1) Walnut-supplemented period had a higher mean total energy consumption vs. control period (8171 kJ (1952) kcal vs. 7614 kJ (1819) kcal, *P* < 0.05). 2) NS in mean ± SEM body weight.
Jaceldo-Siegl K, 2004 USA	To examine the effect of a daily supplement of nuts on the overall habitual diets of healthy subjects. Ages: 25-50 yrs Total n completed = 71	RCT for 12 months. 1) First 6 months was the control period: Habitual diet. 2) Intervention: 6 months of almond supplement (equivalent to 15 % of each subject’s mean energy intake during the habitual diet period; range of intakes: 42-71 g.	24 h telephone diet recalls during each diet period	1) Almond supplementation improved nutrient intakes (monounsaturated fatty acids, 42 %; polyunsaturated fatty acids, 24 %; fibre, 12 %; vegetable protein, 19 %; α-tocopherol, 66 %; magnesium, 23 % (all *P* < 0.05). 2) Almond supplementation decreased the intakes of trans fatty acids, 14 %; animal protein, 9 %; sodium, 21 %; cholesterol 17 %; and sugars, 13 % (all *P* < 0.05).
Johnston C, 2013 USA	1) To examine the long term satiating effect of daily peanut ingestion (28 g/d) on BMI over an 8-week period in overweight adults Ages: 20–65 yrs Total n completed = 44	RCT for 8 weeks. 1) Peanuts (1 oz/28 g) 2) Grain bar (1.4 oz/40 g) Consume the test food 60 minutes prior to the dinner meal daily.	3 d diet records	1) Greater decrease in body weight in the grain bar vs. peanut group (−1.3 ± 0.4 kg vs. −0.2 ± 0.3 kg P = 0.033). 2) NS change in body fat percentage between groups (grain vs. peanut: −1.6 ± 0.5 % vs. −0.5 ± 0.3 % respectively, P = 0.089). 3) NS in mean changes in protein (Δ + 7 ± 6 g vs. Δ −1 ± 5 g, P = 0.22) and fiber (0.2 ± 1.6 vs. 1 ± 2 g/d, P = 0.67) intakes between groups.
Kirk TR, 1997 UK	To test the hypothesis that increased consumption of foods rich in carbohydrate in the form of starch, such as breakfast cereals, will enable a substantial reduction in the percentage dietary energy derived from fat, without any adverse dietary effects. Aged 17-30 years with normal body weight Total n completed = 59	RCT for 12 week. 1) Increase consumption RTEC by 420 g per week (60 g or 2 portions per day, to be taken with semi-skimmed milk). No other dietary advice given. 2) No dietary advice but contact was maintained on a regular basis to arrange dietary and other assessments.	7 d weighed intakes	1) NS between groups in the change in body weight or BMI at 4 weeks or at 12 weeks vs. baseline (-1.4 kg vs. +0.3 kg). 2) NS change in energy intake between the groups. 3) Lower decrease in % total fat (-5.5 % vs. -1.4 %, *P* < 0.05) and higher protein intake (1.4 g/d vs. -3.5 g/d, *P* < 0.001) in intervention vs. control. 4) Greater decrease in % contribution of biscuits and cakes (-6.0 % E vs.-1.4 % E, *P* < 0.05) to daily energy intake.
Rosado J, 2008 Mexico	1) To determine if an increase in RTEC intake is an effective strategy to reduce excess body weight and blood lipids in overweight or at risk of overweight children. 2) To determine if a nutrition education program would make a difference on the response to an increase in cereal intake. 3) To determine if increase in RTEC intake alone or with a nutrition education program has an effect on plasma lipid profile. Ages: 6-12 yrs who were overweight (>85^th^ percentile) or at risk of overweight. Total n completed = 178	RCT for 12 weeks. 1) One serving of 33 ± 7 g of RTEC at breakfast. 2) Two servings of 33 ± 7 g of RTEC, one at breakfast and another serving at dinner. 3) One serving of 33 ± 7 g RTEC and in addition, both children and mothers received a nutrition education guide that contained recommendations for healthy eating. 4) No treatment. 4 options of corn based RTEC, a pre-sweetened corn based RTEC, a pre-sweetened corn based, chocolate flavoured RTEC, and a pre-sweetened rice based, chocolate flavored RTEC.	1) Anthropometry 2) Body composition	Only the children that received 33 ± 7 g of RTEC and nutrition education had: 1) Lower mean (95 % CI) body weight (-1.01 (-1.69, -0.34) kg vs. control (+1.19 (0.39, 1.98) kg, *P* < 0.01) 2) Lower BMI (-0.95 (-1.71, -0.20) kg/m^2^vs. control +0.01 (-0.38, 0.41) kg/m^2^, *P* < 0.01) 3) Lower total body fat % (-0.71 (-1.71, 0.28) %, vs. control (+0.44 (-0.46, 1.35) %, *P* < 0.05) 2) The groups consuming one or two servings of RTEC had no effect on body weight.
Matthews A, 2012 UK	To determine the effects of consuming a structured post-dinner snack in the form of RTEC in place of a normal evening snack on body weight and anthropometric measurements in habitual evening snackers. Ages: 18–55 yrs (mean 40 yrs) Total n completed = 36 in cereal group; *n* = 34 in control group	Randomized, controlled, parallel 6-week intervention study: 1) Intervention: Consume a selection of breakfast cereals at home, and were asked to consume one bowl of cereal (>25 g but <45 g) with 125 ml of semi-skimmed milk after their evening meal, in place of their usual evening snack. 2) Control: Maintain usual dietary and exercise habits.	1) 3 d food diary 2) Anthropometry	1) Evening energy intake was higher in the control vs. treatment group (1259 ± 216 kJ vs. 786 ± 60 kJ, *P* = 0.007). 2) NS between groups in anthropometric measurements. 3) Trend for higher daily energy intake in control vs. treatment group (10,937 ± 1875 kJ vs. 10,014 ± 331 kJ, *P* = 0.065).
*Acute studies*				
Farajian P, 2010 Greece	To test the hypothesis that a preload including dried prunes consumed as a snack before a meal, compared to an isoenergetic bread product preload, would reduce: a) meal time energy intake, b) appetite for dessert offered after lunch and, c) energy intake for the next 24 h. Ages: 18-50 yrs (mean 28 yrs) Total n completed = 45	Randomized within-subject crossover design. Standardised breakfast and then a preload: Standardised lunch and dessert offered 3 hours after the snack. 1) Prunes (i.e. 30 g white bread, 30 g of low-fat (10 % fat) cheese, 5 prunes (40 g): Total 238 cal (1000 kJ). 2) Bread (70 g white bread, 30 g of low-fat (10 % fat) cheese: 244 cal (1025 kJ).	Weighed food before and after eating	1) Total energy intake at the meal (i.e. from lunch and dessert) was lower with prunes preload vs. bread preload (910 ± 233 kcal (3.82 ± 0.98 MJ) vs. 971 ± 249 kcal (4.08 ± 1.04 MJ), *P* = 0.010). 2) NS in energy intake 24 h following the consumption of lunch (Prunes: 1350 ± 386 kcal (5.67 ± 1.62 MJ) vs. Bread: 1450 ± 524 kcal (6.1 ± 2.2 MJ), *P* = 0.021).
Davy BM, 2008	To determine whether pre-meal water consumption reduces meal energy intake in overweight and obese older adults Ages: 55-75 yrs (mean 61.3 yrs) Total n completed = 24 overweight males and females	Randomized trial. Each participant consumed two breakfast meals in a random order: 1) 30-minute waiting period (no preload) followed by an *ad-libitum* standardized meal. 2) Preload consisting of 500 ml of chilled (5° to 7 °C) bottled water, given 30 minutes before an *ad-libitum* standardized meal.	1) Weighed food before and after eating 2) Body weight	1) Gram weight of food consumed at the test meals was less in the water preload vs.no-preload (611 ± 31 g vs. 663 ± 36 g, respectively, *P* = 0.023) 2) Participants consumed less energy at the test meal after the water preload vs. no-preload (74 ± 23 kcal; ~13 % reduction in meal energy intake).
Van Walleghen EL, 2007 USA	To determine whether the consumption of water 30 minutes before an *ad-libitum* meal reduces meal energy intake in non-obese young (and older, mean age 68 yrs) adults. Ages: 21-35 yrs Total n completed = 29	Subjects provided *ad-libitum* lunch meal on two occasions. Thirty minutes before the lunch meals, subjects were given: 1) Water preload (375 mL, women; 500 mL, men). 2) No preload.	1) Weighed food before and after eating	NS in meal energy intake between no preload and water preload in the young subjects (892 ± 51 kcal vs. 913 ± 54 kcal, *P* = 0.65).
Bertenshaw E, 2008 UK	1) To compare appetitive responses (hunger and fullness and subsequent intake) to carbohydrate and protein-enriched drinks administered at 30 min and also 120 min prior to lunch. 2) To observe if the relative satiating efficiency of protein and carbohydrate changes with preload time interval, specifically by impacting energy adjustment. Ages: 18-34 yrs (mean 23 yrs) Total n completed = 18 males	Counterbalanced single blind within-subjects design, with each participant attending six test sessions in total over a 3-week period with a minimum of 2 days between each session. 1) Control test drink (a low-energy apricot and peach fruit drink). 2) Carbohydrate test drink (a higher energy version of the apricot and peach control drink, 97.6 % E carbohydrate). 3) Protein test drink (a higher energy version of the apricot and peach control drink, 50 % protein). 300 ml drinks administered at two time intervals of 120 min and 30 min before the ad libitum test meal. Other meals provided (breakfast, snack, pre-lunch).	Weighed food before and after eating	1) Less food was consumed following the protein vs. carbohydrate preload [F(1,17) = 6.70, *P* < 0.05] and control [F(1,17) = 5.83, *P* < 0.05] preloads. 2) Total caloric intake was significantly higher (+710 kJ) with protein preload vs. control. 3) Carbohydrate preload increased overall energy intake vs.control (+1045 kJ) [F(1,17) = 67.22, *P* < 0.0005], and protein (+334 kJ) [F(1,17) = 5.54, *P* < 0.05].
Flood JE, 2007 USA	To examine further the effects of consuming different forms of a low-energy-dense soup as a preload on subsequent test meal intake and total energy intake at the meal (soup preload + test meal). Ages: 18-45 yrs (mean 26 yrs) Total n completed = 60	Subjects came to the laboratory for lunch once a week for 5 weeks. Each week, one of four compulsory preload soups containing the same energy density (0.33 kcal/g; 1.4 kJ/g), or no preload, was consumed prior to lunch (pasta and sauce). One and a half (350 ml) of soup was served to women, and two cups (475 ml) of soup was served to men. A test meal was consumed *ad-libitum* 15 min after the soup was served: 1) Broth and vegetables served separately. 2) Chunky vegetable soup. 3) Chunky-pureed vegetable soup. 4) Pureed vegetable soup.	Weighed food before and after eating	1) When soup was consumed, subjects reduced lunch meal energy intake by 20 % (~824 kcal, 3.4 MJ) vs. 936 kcal, 3.9 MJ), *P* < 0.0001. 2) NS in energy intake between type of soup consumed. 3) Mean total meal energy density was lower when a soup preload was consumed (1.0 kcal/g, 4.2 kJ/g) vs. no soup (2.2 kcal/g, 9.2 kJ/g).
Rolls BJ, 2010 USA	To investigate the effects on food and energy intakes of varying the portion size and energy density of a vegetable that was added to a meal or substituted for other foods. Ages: 20-45 yrs (mean 27 yrs) Total n completed = 49 in the addition study.	Crossover design with repeated measures. Two studies. In both studies, a midday meal of a vegetable, grain, and meat was served to participants once a week. Across the meals, the vegetable was increased in portion size (180, 270, or 360 g) and reduced in energy density (from 0.8 to 0.4 kcal/g). Addition study: as the vegetable portion was increased, the amounts of the meat and grain were not changed (i.e. total amount of food served at the meal was increased).	Weighed food before and after eating	1) Increasing the portion of the vegetable from 180 to 270 g increased vegetable intake in both studies by a mean ± SE of 34 ± 4 g, equivalent to about half a serving. 2) Doubling the portion of the vegetable (180 to 360 g) increased vegetable intake by 60 ± 5 g (49 ± 4 %). 3) Addition study: Intake of the meat and grain did not differ as the vegetable portion size was increased; there was no significant change in energy intake from the meat and grain and no significant difference in total energy intake at the meal.

*BMI* body mass index, *n* number of participants, *NS* not significant, *RCT* randomized controlled trial, *RTEC* ready to eat cereals, *SE* standard error, *SEM* standard error of the mean, *yrs* years of age, *%E* percentage of energy

**Table 5:** Description of included nutrition education/messages studies

Reference	Study aims	Intervention type, comparator and duration	Outcome measurement	Main results
Sichieri R, 2008 Brazil	To determine whether an educational programme aimed at discouraging students from drinking sugar-sweetened beverages could prevent excessive weight gain. Ages: 9–12 yrs Total n completed = 1140	RCT for 7 months. 1) Healthy lifestyle education programme: Simple messages encouraging water consumption instead of sugar-sweetened carbonated beverages, plus 10 × 1-hour sessions of activities facilitated by four trained research assistants: 20–30 min where teachers were encouraged to reiterate the message during their lesson. 2) 2 × 1-hour general sessions on health issues and printed general advices regarding healthy diets.	1 × 24 h recall	1) NS mean (95 % CI) change in weight or BMI between intervention and control (Δ: weight 2.8 (2.5, 3.2) kg vs. 2.8 (2.6, 3.0) kg; Δ BMI: 0.32 (0.19, 0.46) kg/m^2^ vs. 0.22 (0.13, 0.32 kg/m^2^). 2) Carbonated beverage intake reduced in the intervention vs. control (mean (95 % CI: change: 269.0 (2114, 224) ml/d vs. 213 (256, 31) ml/d). 3) Fruit juice consumption NS increased in intervention group (*P* = 0.08).
Alinia S, 2011 Denmark	1) To investigate the feasibility of using workplaces to increase the fruit consumption of participants by increasing fruit availability and accessibility by a minimal fruit programme. 2) To investigate whether a potential increase in fruit intake would affect vegetable, total energy and nutrient intake. Ages: ~46 yrs Total n completed = 5 workplaces as intervention (*n* = 68), 3 as control (*n* = 56)	5 month, controlled, workplace study. 1) Fruit programme: Fruit basket set out in a room to which participants had free and easy access, such as the reception or the staff kitchen. At least one piece of fruit was available per participant per day. 2) Control: No free fruit.	2 × 24 h dietary recalls	1) Mean ± SE daily fruit consumption increased in intervention vs. control (Δ +112 ± 35 g/d vs. +10 ± 24 g/d, P = 0.021). 2) Mean ± SE intake of dietary fibre increased in intervention vs. baseline (Δ + 3.0 ± 1.1 g/d, P = 0.007), however the change in fibre intake in control was not different to baseline (Δ 0.7 ± 1.0 g/d, *P* > 0.05). 3) Mean ± SE intake of sugar decreased in intervention vs. baseline (Δ -10.7 ± 4.4 g/d, P = 0.019) however the decrease in sugar intake in the control group was not different to baseline (Δ -5.1 ± 4.4 g/d, *P* > 0.05). 4) Mean daily intakes of vegetables, total energy and macronutrients remained unchanged in the intervention group. 4) Only the change in fruit intake was significantly different between the intervention group and the control group (112 g vs. 10 g, *P* = 0.021).
Moore L, 2008 UK	To estimate the impact of school fruit tuck shops on children’s consumption of fruit and sweet and savoury snacks. Ages: Year 5 and Year 6 children (9-11 yrs) Total n completed = 23 intervention schools (*n* = 921); 20 control schools (*n* = 691)	Cluster randomized effectiveness trial (school as the unit of randomization). 1) Schools operated fruit tuck shops throughout one academic year. 2) No tuck shop (control schools).	1) 1 × 24 h recall 2) 1 × 1-y follow-up questionnaire on food preferences	1) NS in fruit intake between intervention and control from fruit at school (0.74 servings vs. 0.69 servings). 2) NS in total daily fruit intake between intervention and control (2.54 servings vs. 2.51 servings). 3) NS in consumption of other snacks between groups. 4) Schools with a ‘no food’ or ‘fruit only’ policy: less fruit consumed vs. schools with no restrictions (mean (95 % CI): 0.37 portions (0.11, 0.64) greater consumption in schools with a fruit only policy; 0.14 (-0.30, 0.58) with a no food policy, and -0.13 (-0.33, 0.07) where there were no restrictions.
Robinson E, 2013 UK	To examine whether a health message and a social norm message about limiting junk food intake would motivate people to reduce their intake of high calorie snack food (a type of junk food at a snack buffet). Ages: Mean age ~23 yrs Total n completed = 39 in social norm; 48 in health, and 42 in control	A 3 × 2 between-subjects design, with factors: message type (social norm/health/control) and usual junk food intake (low consumers/high consumers). In the social norm and health conditions, participants viewed a poster containing images of junk food (a hamburger, fries, soda, candy wrappers) and a message defining junk food: ‘junk food is high calorie food with low nutritional value. The posters only differed in the content of a message in the middle of the poster: 1) Social norm: ‘Students eat less junk food than you might realise. Most students limit how much junk food they are eating to 1 or less than 1 serving/d (based on a 2012 study). 2) Health condition: ‘Reducing junk food intake is good for your health. Limiting junk food to 1 or less than 1 serving a day is part of a healthy diet (based on a 2012 study). 3) Control: Message emphasised the importance of preparing in Ladvance for exams.	Guided one day dietary recall measure (over 24 h) The snack buffet consisted of 6 common food items in the UK (3x high calorie snack foods, plus fruit and vegetable items)	1) High calorie snack food consumed was lower in both the health and the social norm message condition compared with the control message condition (36 % and 28 %, both *P* < 0.05) social norm: 30 (21) g vs. 23 (20) g vs. 42 (38) g, *P* < 0.05). 2) NS for fruit and vegetable intake (social normal: 103 (74) g vs. health: 85 (58) g vs. control: 970 (63) g, *P* > 0.05). 3) NS for total snack intake in social norm (207 (122) kcal) but health condition decreased snack intake (165 (103) kcal vs. control: 266 (210 kcal), *P* < 0.05).

*BMI* body mass index, *CI* confidence interval, *n* number of participants, *NS* not significant, RCT randomized controlled trial, *SE* standard error, *yrs* years of age

## Review

The initial search retrieved 3283 articles (with removal of duplicates), 3181 were excluded through title and abstract screening (Fig. 1). One hundred and two full text articles were examined; following exclusion, 44 studies were included in the review. Thirteen studies assessed reformulation strategies (Table 1), five studies assessed substitution strategies (Table 2), nine studies assessed restriction/elimination strategies (Table 3) and 13 studies assessed supplementation strategies (Table 4). Four studies assessed nutrition education/messages strategies (Table 5).

### Reformulation strategies

#### Chronic dietary manipulation studies

Six RCTs assessed the effect of food reformulation over a chronic period (Table 1). Two of the studies reported positive findings, three studies reported null findings, and two studies reported mixed findings. Gatenby et al. [20] found that in adults, using low fat foods rather than high fat foods *ad-libitum* over 6 weeks, reduced dietary percentage of energy from fat (8 % absolute) and decreased body weight (700 g difference). A further study in this group [21] utilised the same intervention in females only, but also included another arm of low sugar rather than regular sugar foods. Over 10 weeks, there was no change in energy or sugar intake; however, the percentage of energy from total fat was 4 % lower in the low fat group compared to the low sugar and usual food groups [21].

Two studies reported on altering dairy foods [22, 23]: Golley et al. [22] showed that using reduced fat rather than regular fat versions of dairy foods for 12 weeks reduced children’s fat and saturated fat consumption but not total energy intake. Gunther et al. [23] showed in young women that manipulating dairy products with either moderate or high fat dairy whilst maintaining isocaloric intake had no effect on body weight.

Two RCTs reported on manipulating beverage consumption. In adolescents, Ebbeling et al. [24] showed that compared to usual beverage consumption, consuming non-caloric beverages for 25 weeks led to a 1 MJ lower energy intake; however, there was no change in BMI between groups. In overweight adults, Raben et al. [25] compared *ad-libitum* food intake and body weight following 10 weeks of supplemental drinks containing either sucrose or artificial sweetener. It was found that those in the sucrose group tripled their energy intake from sucrose drinks, leading to a >2.5 MJ higher overall total daily energy intake. The sucrose drink group gained 1.6 kg over the 10 weeks compared to a 1 kg reduction in weight in the artificial sweetener group [25].

#### Acute studies

Acute reformulation strategies were assessed in seven studies (Table 1). In young children, consumption of plain milk (256 kJ/100 mL), sucrose-sweetened chocolate milk (416 kJ/100 mL), or aspartame-sweetened chocolate milk (263 kJ/100 mL) had no significant effect on the consumption of other food items offered at that meal [26]. However, children consuming high-sugar ready-to-eat cereal (RTEC) consumed almost twice the amount of refined sugar at the breakfast meal (from RTEC and added sugar: 24.4 g vs. 12.5 g) and consumed a greater quantity of RTEC compared to children consuming the lower sugar RTEC (61 g vs. 35 g) [27].

In adults, provision of snacks enriched with either barley beta-glucan [28] or different macronutrient composition [29, 30] had no impact on subsequent food or energy intake. Manipulating the energy density of a meal decreased meal intake in children [31], however, keeping the same energy density but increasing the air content of a snack food reduced energy intake of the snack but not subsequent snack energy intake [32].

### Substitution strategies

#### Chronic studies

Two studies reported on substitution as a strategy to improve nutrient/food intake and body weight. Substituting high fibre snacks (10–12 g/d fibre) for 8 weeks in children [33] or substituting cereal bars (high in carbohydrate) or almonds (high in protein) for 12 weeks in adults [34] in place of usual snacks, had no effect on macronutrient intakes [33, 34] or body weight [34] (Table 2). Consumption of high fibre snacks in children also had no effect on fibre or whole grain intake; however, intake of total grains, and sweets, was higher [33].

#### Acute studies

Three studies reported on the acute effects of substituting lower energy foods/beverages in a main meal or snack (Table 2). In adults, Flood et al. [35] substituted regular calorie with low or no calorie beverages using a variety of portion sizes. It was found that when a larger portion of beverage was served (540 g vs. 260 g), more energy from the beverage was consumed (~100 kJ); however, there was no difference in the overall meal energy intake [35]. Including extra vegetables into a meal while reducing the portion size of meat and grain, increased vegetable intake (~0.75 servings) but also decreased energy intake from the meat and grain component, and therefore the overall meal [36]. In children, an afternoon snack consisting of either raisins or grapes reduced subsequent dinner energy intake by 200–300 kJ compared to a snack of energy dense potato chips or chocolate chip cookies [37].

### Restriction/elimination strategies

#### Acute studies

Nine studies reported on restriction/elimination strategies via manipulating portion sizes of entrées, main meals or snacks (Table 3). Increasing the portion size of a salad entrée reduced subsequent main meal intake by 410 kJ in adults [38]; while quadrupling the portion size of macaroni and cheese (energy density of 1.52 kcal/g (6.36 kJ/g)) within a meal (i.e. 100 g vs. 400 g, with an energy density of 1.12 kcal/g vs. 1.27 kcal/g) increased intake of macaroni and cheese by 150 %, and total meal intake by 61 % [39] in children. This supports the findings of the previous study by Fisher et al. [40] that found doubling the portion size (250 g vs. 500 g) of macaroni and cheese (1.42 kcal/g) in a meal increased macaroni and cheese intake by 33 % and total meal intake by 15 % in pre-school children.

In adolescents, serving the same take-away meal in different portion sizes (4 smaller servings presented at a single time point vs. portioned into 4 smaller servings presented at 15-min intervals) had no effect on overall take-away food intake compared to the meal served in one large portion [41]. In younger children, a large portion size of a low calorie snack (applesauce) or high calorie snack (chocolate pudding) increased subsequent meal energy intake compared to a smaller portion size of the same foods [42].

In children, halving the item size of candies [43] or cookies [44] led to a reduction in intake of 60 kcal and 68 kcal, respectively. In adults, consuming portion controlled 100 kcal snack packs (19 g–26 g) of crisps, crackers, pretzels or cookies led to a weekly caloric deficit of 841 kcal/week compared to larger, usual snack packs (187 g–369 g) [45]; while consuming smaller portions of a sandwich (6, 8, 10, or 12 in.) also reduced energy intake [46].

### Supplementation strategies

#### Chronic studies

Seven chronic studies reported on the effect of supplementing the diet with specific foods/beverages on micronutrient intake or body weight, in which the majority of studies reported null results (Table 4). Supplementing 43 g/d of almonds for 4 weeks in adults with type 2 diabetes [47] or walnuts for 12 weeks [48] did not affect micronutrient intake [47], body weight [47, 48] or body composition [48]. Six months of almond consumption (range of intakes: 42–71 g/d) significantly increased micronutrient intakes and decreased intakes of sodium and sugars by more than 10 % [49].

Consumption of a grain bar high in carbohydrate prior to the evening meal for 8 weeks decreased body weight compared to consumption of a peanut bar high in protein (mean difference 1.5 kg), yet there was no difference between groups in reported energy or protein intake [50]. Consuming RTEC in amounts ranging from 33–60 g/d did not consistently improve dietary intake or anthropometric measures [51–53], and these studies did not report whether and what types of foods RTEC supplemented, except in the study by Kirk et al. [51] who supplemented bread and toast. The study by Kirk et al. [51] also reported a greater decrease in the percent contribution of biscuits and cakes to mean daily energy intake compared with the control group who received no advice to consume RTEC for 12 weeks (-6.0 %E vs -1.4 %E); one or two servings of RTEC for 12 weeks did not affect body weight in children [53]; but when RTEC and milk was consumed in place of the usual evening snack (food type not reported), an increase in evening energy intake (+500 kJ) was found [52].

#### Acute studies

Six studies reported on supplementation using food or beverage pre-loads (low calorie food or beverage) before or within a meal, where most studies reported a reduction in meal energy intake (Table 4). In adults, a snack of prunes before a meal reduced subsequent energy intake at the meal compared to a snack of white bread, however the reduced energy intake did not remain over a 24-h period [54]. A 500 ml water pre-load significantly reduced meal energy intake by 13 % [55] and 8 % [56] in older adults; however a 375 ml or 500 ml water pre-load in younger women and men did not alter meal and energy intake [56]. Comparatively, consumption of an apricot and peach drink high in protein led to a reduction in subsequent test meal food intake in young adults compared to the same apricot and peach drink that was higher in carbohydrate, or a low energy control version [57].

In adults, consumption of 1.5–2 cups of soup in a variety of flavours (with the same energy density) before a meal, reduced meal energy intake by 20 %; however the type of soup had no significant effect on test meal intake or total meal energy intake [58]. In the same study described earlier, Rolls et al. [36] also showed that including extra vegetables into a meal without reducing the portion size of the meat and grain component increased vegetable intake (~0.75 servings); however the addition of more vegetables did not significantly affect meal energy intake.

### Nutrition education/messages strategies

Four discrete intervention studies were identified that assessed nutrition education/messages strategies to improve nutritional intake and/or decrease discretionary choices (Table 5). The study by Sichieri et al. [59] encouraged simple messages “to consume water instead of sugar-sweetened beverages” in 9–12 year olds over 7 months. Overall, there was a significant reduction in carbonated beverage intake (~390 ml/d) compared with the control group who received no information on substitution. However, this did not equate to a reduction in weight or BMI, potentially due to slight increases in fruit juice intake in both groups (35–84 ml/week). In the study by Alinia et al. [60] free access to a fruit basket for 5 months at various workplaces increased the consumption of fruit by nearly 1 serving/d vs control; intake of dietary fibre increased by 3 g/d and added sugar decreased by 2 teaspoons/d in the intervention group vs baseline. In contrast, provision of a fruit tuck shop in primary and junior schools in the UK had no effect on increasing fruit intake over the 9 month period [61]. However, in the schools with a ‘no food’ or ‘fruit only’ policy, the fruit tuck shop intervention had a greater impact than in schools with no restrictions, whereby there was 0.37 portions greater consumption of fruit in schools with a fruit only policy compared with 0.14 portions with a no food policy and -0.13 where there were no restrictions [61].

The final study assessed positive and negative nutrition messages on food intake [62]. Young adults who read messages about the health effects of junk food (i.e. “reducing junk food intake is good for your health”) or social expectations (i.e. “students eat less junk food than you might realise”) consumed fewer high calorie snack foods compared with those who read messages unrelated to junk food consumption; however, there was no effect on fruit and vegetable intakes between groups [62].

## Discussion

This scoping review assessed the impact of discrete dietary manipulation strategies that were potentially applicable to reducing discretionary choices in adults and children. Although no definitively effective single discrete strategy was identified, there were a number that show potential, including reducing portion size, reformulation of fat (from higher to lower saturated fat), substituting high fibre snacks or low/no-caloric beverages into the diet, supplementing healthy nutrient dense foods such as nuts and wholegrain cereals, using a combination of permissive and restrictive education messages, and incorporating cheap and/or complimentary healthier foods in the workplace. The findings of this review can inform future research, in particular adapting these strategies in the development of interventions to reduce excess intake of discretionary choices.

A limited number of chronic dietary manipulation studies addressed the use of reformulation as a strategy to improve diet quality. While a change from regular fat foods to reduced fat foods appears to be a useful strategy in terms of lowering fat intake [20, 21], the impact on overall energy intake and bodyweight are not clear [23]. Consumption of non-caloric beverages appears to reduce overall energy intake in adolescents [24] and adults [25], however reductions in body weight tend to occur only in adults.

Short-term consumption of sucrose compared to artificially sweetened milk did not alter subsequent food intake in children [26], and consuming snacks with different macronutrient profiles [28–30] or consuming a more-aerated snack [32] had no effect on subsequent meal energy intake. Nevertheless, since a more aerated snack did result in less energy consumed from the snack, and reformulating energy dense meals for lower density versions reduced energy intake at that meal [31], manipulating food density has potential for reducing discretionary choices and should be investigated further.

There were mixed results regarding substitution strategies. Chronic studies substituting high fibre snacks [33], cereal bars or almonds [34] for usual snacks did not improve micronutrient intake, and high fibre snacks in children actually increased intake of sweets. Interestingly, in the high fiber snack study by Brachula et al. [33] participants in the intervention group did not usually consume snacks. Thus incorporating two eating occasions of a high fiber snack to their usual routine, with no change in total grain but an increase in whole grain intake, indicates that the children were likely displacing refined grains and hence discretionary choices, and therefore undertaking a positive behavior change.

Smaller portions of non-caloric beverages reduced meal energy intake [35], and increasing vegetable portions within a meal increased vegetable consumption and decreased meat and grain intake [36]. Reducing energy density of foods and beverages appears to be an appropriate strategy, at least in the short term, for reducing energy intake. This approach requires investigation in contexts when the meal consists heavily of discretionary choices, such as fast food outlets, to review the impact on reducing energy intake and improving nutrient intake. Studies assessing low/no-caloric beverages or raisins as a substitute for high calorie beverages and crisps/cookies, respectively, suggest these may be effective strategies for displacing discretionary choices. The long-term impact of such strategies remains to be evaluated.

Most restriction/elimination studies were effective in reducing short-term (within-meal) energy intake. These studies typically reduced the portion size of specific meal components or increased the portion of a low-density meal (e.g. a salad), which subsequently reduced energy density and energy intake [38–40, 42]. The theory behind this is that lower energy density foods might displace intake of some of the higher energy-density foods at a meal or at a subsequent meal, such as discretionary choices, through its satiating effect [38, 63]. However, one study showed that reducing the energy content of various entrées led to an increase in consumption of discretionary foods, and while this resulted in a decrease in daily energy intake, ratings of hunger were increased [64]. Increased hunger could translate into increased consumption of discretionary choices. Large portions of low energy dense foods, which provide a feeling of fullness, may be a more effective strategy to moderate energy intake and decrease discretionary choices at a meal or between meals. Halving item sizes of candies [43] or cookies [44], or reducing portion sizes of discretionary choice snacks [45], or a sandwich [46] was also an effective strategy for reducing energy intake. Although these are effective in an acute setting, the longer-term effects of restricting portion or snack item sizes should be investigated in the context of reducing subsequent discretionary choices and energy intake.

Results were mixed for the effect of chronic supplementation strategies on nutrient profile and body weight [47–53]. Given the nutrient dense profile of nuts and wholegrain foods, supplementing the diet with these foods compared to discretionary choices would support overall diet quality. The net benefits of supplementing the diet with RTEC are less clear since many are high in added sugar and salt.

Acute supplementation studies generally have a positive effect on reducing subsequent meal energy intake and could be a strategy to reduce discretionary choices after a main meal. A water pre-load could be a useful strategy to decrease energy intake in older adults who are overweight or obese, however its effect on replacing or reducing higher calorie foods in the diet requires further investigation, so too does its effect in non-obese younger and older adults. Including low calorie, non-energy dense meals prior to a discretionary choices main meal has a beneficial impact on subsequent meal energy intake.

Free access to fruit is a positive strategy to increase fruit and fibre intake, and reduce added sugar intake in adults [60], and therefore potentially discretionary choices; however, the impact of this in children was ineffective and requires nutrition policies within the school to produce any positive effect [61]. Interestingly, the effect in adults occurred without any guidance or nutrition education and only with the provision of a free fruit basket at the office workplace, yet this was only one study and therefore requires further investigation. Nevertheless, differences in outcomes/effects between these studies may be the result of older adults having a better understanding on the benefits of fruit intake compared to younger children; possibly lower accessibility of fruit at a tuck shop where students are required to get the fruit themselves compared to having it in the classroom; or potential problems in the validity and reliability of self-reported fruit intake between adults and children.

Only a limited number of studies utilised nutrition messages to change nutritional intake. Permissive messages to increase water consumption are beneficial to reducing sugar sweetened beverage intake [59] and restrictive messages about the impact of junk food on health and its social acceptance, also appears to reduce discretionary food intake [62]. However, the longer-term implications of these messages are unknown.

Strengths and limitations to this study are acknowledged. This review is the first to comprehensively investigate the impact of discrete dietary strategies that were potentially applicable to reducing discretionary choices, on discretionary choices intake and/or the strategies targeted health/nutrition effects. The review captured a wide range of studies as the inclusion criteria was broad and was not limited by age; and the search was extensive by way of multiple databases. Although we relied on published content and did not contact authors or search grey literature, the purpose of a scoping review is to be broader than a systematic review so that key findings can be later assessed in a systematic process. Limitations include the small number of studies that utilized similar dietary strategies, thus drawing firm conclusions on study outcomes/effects was difficult; and although not a key feature of scoping review [16] we did not assess study bias, however this review is transparent and has not placed undue emphasis on one study relative to another. As this study did not assess intake of discretionary choices per se for all identified strategies, the impact on this outcome was not always able to be determined; further research is required to investigate how the alternative targeted health/nutrition effects for these strategies are affected by and/or affect intake of discretionary choices. Another limitation is that only one author reviewed the potentially relevant studies; however, initial discussion around inclusion/exclusion criteria and final studies extracted involved all authors, thereby minimizing any error in included studies. Finally, as there was heterogeneity between study populations, it remains unclear whether some strategies are more feasible in particular population groups.

## Conclusions

No single discrete strategy was identified that definitively reduced discretionary choices in adults or children. However, restriction/elimination strategies (specifically reducing portion size) were consistently beneficial for reducing energy intake, at least in the acute setting. Reformulation of fat (from higher to lower fat) could be useful to reduce saturated fat intake, and substituting high fibre snacks, fruit, or low/no-caloric beverages for discretionary choices may be effective. Supplementation strategies where nutrient dense foods such as nuts and wholegrain cereals are consumed in place of discretionary choices support an improved overall diet quality. Regarding education strategies, a combination of permissive and restrictive messages may reach a large audience to effectively modify behavior, while incorporation of cheaper and/or freely accessible healthier foods in the workplace may be a further option to reduce discretionary choices and support diet quality in adults. Longer-term, well-controlled and larger studies are required to confirm the effectiveness of the proposed strategies and assess their impact in multi-component interventions.

## Appendix 1: Search terms

### Ovid: Medline

1. Diet/

2. Eating/

3. Drinking/

4. ((food adj1 intake) or food ingestion or eat$3 or food consumption).mp. [mp = title, abstract, original title, name of substance word, subject heading word, keyword heading word, protocol supplementary concept word, rare disease supplementary concept word, unique identifier]

5. exp Energy Intake/

6. (calorie intake or energy intake).mp. [mp = title, abstract, original title, name of substance word, subject heading word, keyword heading word, protocol supplementary concept word, rare disease supplementary concept word, unique identifier]

7. or/1–6

8. intervention$1.mp. [mp = title, abstract, original title, name of substance word, subject heading word, keyword heading word, protocol supplementary concept word, rare disease supplementary concept word, unique identifier]

9. Randomized Controlled Trials as Topic/

10. randomized controlled trial/

11. Random Allocation/

12. Double Blind Method/

13. Single Blind Method/

14. clinical trial/

15. controlled clinical trial.pt.

16. randomized controlled trial.pt.

17. multicenter study.pt.

18. clinical trial.pt.

19. exp Clinical Trials as topic/

20. (clinical adj trial$).tw.

21. ((singl$ or doubl$ or treb$ or tripl$) adj (blind$3 or mask$3)).tw.

22. randomly allocated.tw.

23. (allocated adj2 random$).tw.

24. systematic review/or systematic review.tw.

25. meta?analys$2.mp. or metaanalysis/[mp = title, abstract, original title, name of substance word, subject heading word, keyword heading word, protocol supplementary concept word, rare disease supplementary concept word, unique identifier]

26. or/8–25

27. (discretionary choice$1 or snack$1 or treat$1 or extra food$1 or non?core food$1 or “sometimes food$1” or energy dense or nutrient poor or EDNP or empty calor$3 or junk food$1 or unhealthy food$1 or soft drink$1 or sugar sweetened beverage$1 or SSB or soda or sugary drink$1 or fast?food$1 or take?away food$1).mp. [mp = title, abstract, original title, name of substance word, subject heading word, keyword heading word, protocol supplementary concept word, rare disease supplementary concept word, unique identifier]

28. (corn chip$1 or potato chip$1 or chocolate$1 or lollies or sweets or cake or yeast bun$1 or sweet biscuit$1 or hamburger$1 or burger$1 or donut$1 or doughnut$1 or fries or crisps or ice?cream$1 or pudding$1 or dessert$1).mp. [mp = title, abstract, original title, name of substance word, subject heading word, keyword heading word, protocol supplementary concept word, rare disease supplementary concept word, unique identifier]

29. exp Dietary Carbohydrates/

30. sugar.mp.

31. exp Sodium, Dietary/

32. salt.mp.

33. saturated fatty acid.mp.

34. (calorie intake or energy intake).mp. [mp = title, abstract, original title, name of substance word, subject heading word, keyword heading word, protocol supplementary concept word, rare disease supplementary concept word, unique identifier]

35. (diet$3 sucrose or high fructose corn syrup).mp. [mp = title, abstract, original title, name of substance word, subject heading word, keyword heading word, protocol supplementary concept word, rare disease supplementary concept word, unique identifier]

36. (diet$3 sodium or sodium chloride, diet$3).mp. [mp = title, abstract, original title, name of substance word, subject heading word, keyword heading word, protocol supplementary concept word, rare disease supplementary concept word, unique identifier]

37. High Fructose Corn Syrup/

38. Dietary Sucrose/

39. Fatty Acids/

40. or/27–39

41. (strateg$3 or behavio?r).mp. [mp = title, abstract, original title, name of substance word, subject heading word, keyword heading word, protocol supplementary concept word, rare disease supplementary concept word, unique identifier]

42. 7 and 26 and 40 and 41

43. limit 42 to (english language and humans)

44. limit 43 to (“preschool child (2 to 5 years)” or “child (6 to 12 years)” or “adolescent (13 to 18 years)” or “young adult and adult (19–24 and 19–44)” or “middle age (45 to 64 years)”)

### Ovid: Embase

1. Diet/

2. Eating/

3. Drinking/

4. ((food adj1 intake) or food ingestion or eat$3 or food consumption).mp. [mp = title, abstract, original title, name of substance word, subject heading word, keyword heading word, protocol supplementary concept word, rare disease supplementary concept word, unique identifier]

5. exp Energy Intake/

6. (calorie intake or energy intake).mp. [mp = title, abstract, original title, name of substance word, subject heading word, keyword heading word, protocol supplementary concept word, rare disease supplementary concept word, unique identifier]

7. or/1–6

8. intervention$1.mp. [mp = title, abstract, original title, name of substance word, subject heading word, keyword heading word, protocol supplementary concept word, rare disease supplementary concept word, unique identifier]

9. Randomized Controlled Trials as Topic/

10. randomized controlled trial/

11. Random Allocation/

12. Double Blind Method/

13. Single Blind Method/

14. clinical trial/

15. (controlled clinical trial or randomzed controlled trial or multicent$2 study or clinical trial).mp. [mp = title, abstract, original title, name of substance word, subject heading word, keyword heading word, protocol supplementary concept word, rare disease supplementary concept word, unique identifier]

16. exp Clinical Trials as topic/

17. (clinical adj trial$).tw.

18. ((singl$ or doubl$ or treb$ or tripl$) adj (blind$3 or mask$3)).tw.

19. randomly allocated.tw.

20. (allocated adj2 random$).tw.

21. systematic review/or systematic review.tw.

22. meta?analys$2.mp. or metaanalysis/[mp = title, abstract, original title, name of substance word, subject heading word, keyword heading word, protocol supplementary concept word, rare disease supplementary concept word, unique identifier]

23. or/8–22

24. (discretionary choice$1 or snack$1 or treat$1 or extra food$1 or non?core food$1 or “sometimes food$1” or energy dense or nutrient poor or EDNP or empty calor$3 or junk food$1 or unhealthy food$1 or soft drink$1 or sugar sweetened beverage$1 or SSB or soda or sugary drink$1 or fast?food$1 or take?away food$1).mp. [mp = title, abstract, original title, name of substance word, subject heading word, keyword heading word, protocol supplementary concept word, rare disease supplementary concept word, unique identifier]

25. exp Dietary Carbohydrates/

26. (corn chip$1 or potato chip$1 or chocolate$1 or lollies or sweets or cake or yeast bun$1 or sweet biscuit$1 or hamburger$1 or burger$1 or donut$1 or doughnut$1 or fries or crisps or ice?cream$1 or pudding$1 or dessert$1).mp. [mp = title, abstract, original title, name of substance word, subject heading word, keyword heading word, protocol supplementary concept word, rare disease supplementary concept word, unique identifier]

27. sugar.mp.

28. exp Sodium, Dietary/

29. salt.mp.

30. saturated fatty acid.mp.

31. (calorie intake or energy intake).mp. [mp = title, abstract, original title, name of substance word, subject heading word, keyword heading word, protocol supplementary concept word, rare disease supplementary concept word, unique identifier]

32. (diet$3 sucrose or high fructose corn syrup).mp. [mp = title, abstract, original title, name of substance word, subject heading word, keyword heading word, protocol supplementary concept word, rare disease supplementary concept word, unique identifier]

33. (diet$3 sodium or sodium chloride, diet$3).mp. [mp = title, abstract, original title, name of substance word, subject heading word, keyword heading word, protocol supplementary concept word, rare disease supplementary concept word, unique identifier]

34. High Fructose Corn Syrup/

35. Dietary Sucrose/

36. Fatty Acids/

37. or/24–36

38. (strateg$3 or behavio?r).mp. [mp = title, abstract, original title, name of substance word, subject heading word, keyword heading word, protocol supplementary concept word, rare disease supplementary concept word, unique identifier]

39. 7 and 23 and 37 and 38

40. limit 39 to embase

41. limit 40 to (human and english language)

42. limit 41 to (preschool child <1 to 6 years > or school child <7 to 12 years > or adolescent <13 to 17 years > or adult <18 to 64 years>)

### Ebscohost: CINAHL

**Subject headings:** Randomized controlled trial OR Random Assignment OR Double Blind Studies OR Single Blind Studies OR Clinical Trials OR Multicenter Studies OR Systematic Review OR Meta Analysis; Food Intake OR Diet OR Eating; Energy intake OR Fatty acids, Saturated, OR Dietary Sucrose OR High Fructose Corn Syrup OR Carbohydrates OR Glucose OR Sodium Chloride OR Salt; Intervention Trials; Eating Behavior

**Keywords:** Double-Blind Studies OR Intervention Trials OR Randomized Controlled Trials OR Single-Blind Studies OR Random Assignment OR Double Blind Studies OR Single Blind Studies OR Clinical Trials OR Phase 1 clinical trial OR Phase 2 clinical trial OR Phase 3 clinical trial OR Phase 4 clinical trial OR Controlled Clinical Trial OR Multicenter Studies OR Systematic Review OR Meta Analysis; Food Intake OR Diet OR Eating OR food ingestion or eat* or food consumption or discretionary choice* or snack* or treat* or extra food* or non#core food* or “sometimes food*” or energy dense or nutrient poor or EDNP or empty calor* or junk food* or unhealthy food* or soft drink* or sugar sweetened beverage* or SSB or soda or sugary drink* or fast#food* or take#away food* or corn chip* or potato chip* or chocolate* or lollies or sweets or cake or yeast bun* or sweet biscuit* or hamburger* or burger* or donut* or doughnut* or fries or crisps or ice#cream* or pudding* or dessert; Energy intake OR Fatty acids, Saturated, OR Dietary Sucrose OR High Fructose Corn Syrup OR Carbohydrates OR Table sugar OR Added sugar OR Glucose OR Sodium Chloride, Dietary OR Salt; Intervention Trials; Eating Behavior OR Strategy

Limit 2–64 years; English language; Academic Journals, Human

### SCOPUS

((TITLE-ABS-KEY(Energy intake OR Saturated Fat OR sugar OR Salt or total fat)) AND (TITLE-ABS-KEY(Food Intake OR Diet OR discretionary choice or snack or treat or extra food or noncore food or energy dense or takeaway food or fast food or junk food or unhealthy food))) AND (TITLE-ABS-KEY(randomized controlled trials)).

### Cochrane Library Database

Energy intake OR Saturated Fat OR sugar OR Salt or total fat: ti,ab,kw and Food Intake OR Diet OR discretionary choice or snack or treat or extra food or noncore food or energy dense or takeaway food or fast food or junk food or unhealthy food: ti,ab,kw and randomized controlled trials (Word variations have been searched).

## Abbreviations

BMI: body mass index
CI: confidence intervals
NS: not significant
RCT: randomized controlled trials
RTEC: ready to eat cereals
SE: standard error
SEM: standard error of the mean
SSB: sugar sweetened beverages
